# Assessing Privacy Vulnerabilities in Genetic Data Sets: Scoping Review

**DOI:** 10.2196/54332

**Published:** 2024-05-27

**Authors:** Mara Thomas, Nuria Mackes, Asad Preuss-Dodhy, Thomas Wieland, Markus Bundschus

**Affiliations:** 1 F. Hoffmann-La Roche AG Basel Switzerland; 2 xValue GmbH Ratingen Germany; 3 Roche Diagnostics GmbH Penzberg Germany; 4 Foundation Medicine GmbH Penzberg Germany

**Keywords:** genetic privacy, privacy, data anonymization, reidentification

## Abstract

**Background:**

Genetic data are widely considered inherently identifiable. However, genetic data sets come in many shapes and sizes, and the feasibility of privacy attacks depends on their specific content. Assessing the reidentification risk of genetic data is complex, yet there is a lack of guidelines or recommendations that support data processors in performing such an evaluation.

**Objective:**

This study aims to gain a comprehensive understanding of the privacy vulnerabilities of genetic data and create a summary that can guide data processors in assessing the privacy risk of genetic data sets.

**Methods:**

We conducted a 2-step search, in which we first identified 21 reviews published between 2017 and 2023 on the topic of genomic privacy and then analyzed all references cited in the reviews (n=1645) to identify 42 unique original research studies that demonstrate a privacy attack on genetic data. We then evaluated the type and components of genetic data exploited for these attacks as well as the effort and resources needed for their implementation and their probability of success.

**Results:**

From our literature review, we derived 9 nonmutually exclusive features of genetic data that are both inherent to any genetic data set and informative about privacy risk: biological modality, experimental assay, data format or level of processing, germline versus somatic variation content, content of single nucleotide polymorphisms, short tandem repeats, aggregated sample measures, structural variants, and rare single nucleotide variants.

**Conclusions:**

On the basis of our literature review, the evaluation of these 9 features covers the great majority of privacy-critical aspects of genetic data and thus provides a foundation and guidance for assessing genetic data risk.

## Introduction

### Privacy Risks of Genetic Data

Genomics is a rapidly developing field with exabytes of genetic data being generated, stored, and analyzed by public and private institutions per year. These data drive scientific progress, especially when they are shared with the scientific community or among institutions. However, genetic data can provide information about an individual’s identity together with sensitive details, such as their ethnic background [1]; physical traits such as eye color [2], hair and skin color [3], height [4]; and diseases or susceptibility to diseases [5]. Therefore, even if personal identifiers (eg, name, date of birth, or others) are removed, sharing genetic data may violate the individual’s right to privacy. In 2018, a seminal study demonstrated that it is possible to reidentify individuals by name from genetic data alone [6]. The authors matched genetic data of an anonymous female study participant to the genetic genealogy database GEDmatch and identified her surname from matches with relatives who had uploaded their data on GEDmatch. Such reidentification of genetic data records using publicly available databases is highly problematic and a growing threat to privacy as publicly available genetic genealogy databases continue to grow. It is estimated that a genetic database needs to cover “only 2% of the target population to provide a third-cousin match to nearly any person” in a matching attack, similar to the one demonstrated by Erlich et al [6]. As of 2018, the probability for such a match was estimated to be 60% for the platform GEDmatch. Through similar methods of familial DNA searches, multiple individuals have been identified in criminal cases, despite never having shared their genetic data themselves [7,8]. Other attacks aim to reveal sensitive information from genetic data. In 2009, researchers discovered a genetic predisposition for Alzheimer disease in the public genome of the famous molecular biologist and Nobel laureate James Watson, although he had attempted to prevent such an attack by withholding certain parts of the data [9]. The high identifiability potential of genetic data together with its sensitive content with regard to health (eg, susceptibility to diseases such as Alzheimer disease or cancer) and physical traits (refer to the studies by Erlich and Narayanan [10], El Emam et al [11], and Mohammed Yakubu and Chen [12] for a review) has raised public concern that genetic data that are shared or published in the context of research or health care could be misused [13]. For example, attackers could exploit genetic data to obtain personal and sensitive information about individuals, and this information could be misused by insurance companies, mortgage providers, or employers to discriminate on the basis of genetic information (eg, about disease susceptibility) [14]. As an additional complication, DNA sequence is heritable; therefore, leakage of an individual’s genetic data can violate the privacy of whole families [15,16].

### The Challenge of Anonymizing Genetic Data

Genetic data can be used to identify individuals because each person’s DNA sequence differs uniquely from the standard human reference genome. Although more than 99% of the DNA sequence is identical across all humans, the remaining <1% consists of distinct combinations of insertions, deletions, duplications, translocations, and inversions of short or long DNA fragments (refer to the study by Trost et al [17] for a review). These genetic variations are not randomly distributed across the genome but occur more frequently in specific variable regions. Some variations are rare, while others (ie, polymorphisms) are shared by a significant proportion of the population. While some variations have no observable effect, others influence gene transcription, expression, or the amino acid sequence of a protein and have an effect on the phenotype, for example, physical traits, metabolism, and disease susceptibility. These variable regions with an effect on the phenotype are of great interest to research; however, these can also be effectively used for individual identification and the inference of sensitive attributes. Even a small genetic data set of only 30 highly variable genetic loci is likely to contain unique records, and these could not only be linked to genetic records in other data sets but also provide insights into health and physical traits (refer to the studies by Erlich and Narayanan [10], El Emam et al [11], and Mohammed Yakubu and Chen [12] for a review). Furthermore, genetic variation is highly intercorrelated (variation in one genomic region correlates with variation in another) and correlated to other modalities (genetic variation is associated with transcription, expression, epigenetic regulation, etc), making it possible to link data records of the same individual even across databases that do not contain the same type of data (eg, match a genetic data sequence to a gene expression record). Anonymizing genetic data while maintaining its full utility remains an unsolved challenge, and there is no consensus on whether it is even possible [18]. Many privacy-enhancing technologies aim to reduce the information content of genetic data or restrict access to it, such that only a minimal amount of information is shared. An example is genomic beacons, which allow only simple yes or no queries to determine whether a specific variant is present in a study cohort [19]. However, it has become evident that even this limited amount of information can be exploited for privacy attacks, and few queries to genomic beacons can suffice to determine whether individuals (whose genome is known) are present in a study cohort [20-23]. Similarly, proposals for encryption and differential privacy approaches [24,25] have often been countered by demonstrations of attacks [26-28], and even synthetic genetic data may not fully protect the study participants from privacy attacks [29] (refer to the study by Mittos et al [30] for a review of privacy-enhancing technologies). Thus, even a substantial reduction in information content can often not completely eliminate all privacy risks of genetic data [31].

### The Risk Minimization Approach for Genetic Data Privacy

Most legislations do not require to reduce the risk of individual identification to zero, and several jurisdictions have decided to take a risk-based approach and consider genetic data anonymous if the risk of successful reidentification is below a predefined acceptable threshold [32]. Therefore, genetic data processors must find the balance between reducing information such that reidentification is no longer reasonably likely, while maintaining as much utility of the data as possible [33]. The challenge in adopting this approach lies in the correct assessment of the reidentification probability. Genetic data are complex and come in various shapes or forms, making it difficult to standardize reidentification assessments. Established methods such as assessing k-anonymity are difficult to apply to genetic data because of their high uniqueness, and many other methods fall short because of the high intercorrelation of genetic data. Simple measures such as assessing the number of single nucleotide polymorphisms (SNPs) in genetic data ignore the importance of the location of the SNPs in the genome, their frequencies in the population, and the actual feasibility of cross-linking the specific SNPs to identifiable information. For example, the reidentification risk is much higher for SNPs that are commonly included in the SNP assays used by direct-to-consumer genetic testing (DTC-GT) providers than for less frequently studied SNPs, as these are more difficult to link to publicly available identifying information. In addition, genetic data may contain SNP information even if this is not immediately evident, for example, in the raw data of sequencing-based gene expression studies. Data processors who are not familiar with the intricacies of genetic data find little guidance on performing an assessment on genetic data that considers these factors. While several genomic privacy metrics have been proposed, the great majority focus on evaluating SNPs only [34] and neglect other known privacy-critical aspects of genetic data as well as aspects of feasibility (eg, the expertise, time, effort, availability of external resources, and other requirements required for an attack). However, the risk of severe privacy attacks on genetic data (ie, where the identity of the data subject is revealed) greatly depends on the specific content of the data as well as “soft factors,” such as the availability of publicly accessible resources to cross-link and infer quasi-identifying information and the time, cost, and knowledge required to perform such an attack. Given the foundational potential of genetic data to advance research and health care, a risk-based approach that carefully evaluates the true risk of reidentification on a case-by-case basis for each data set in question is warranted, or else any type of genetic data must be considered identifiable.

## Methods

**Figure 1:**
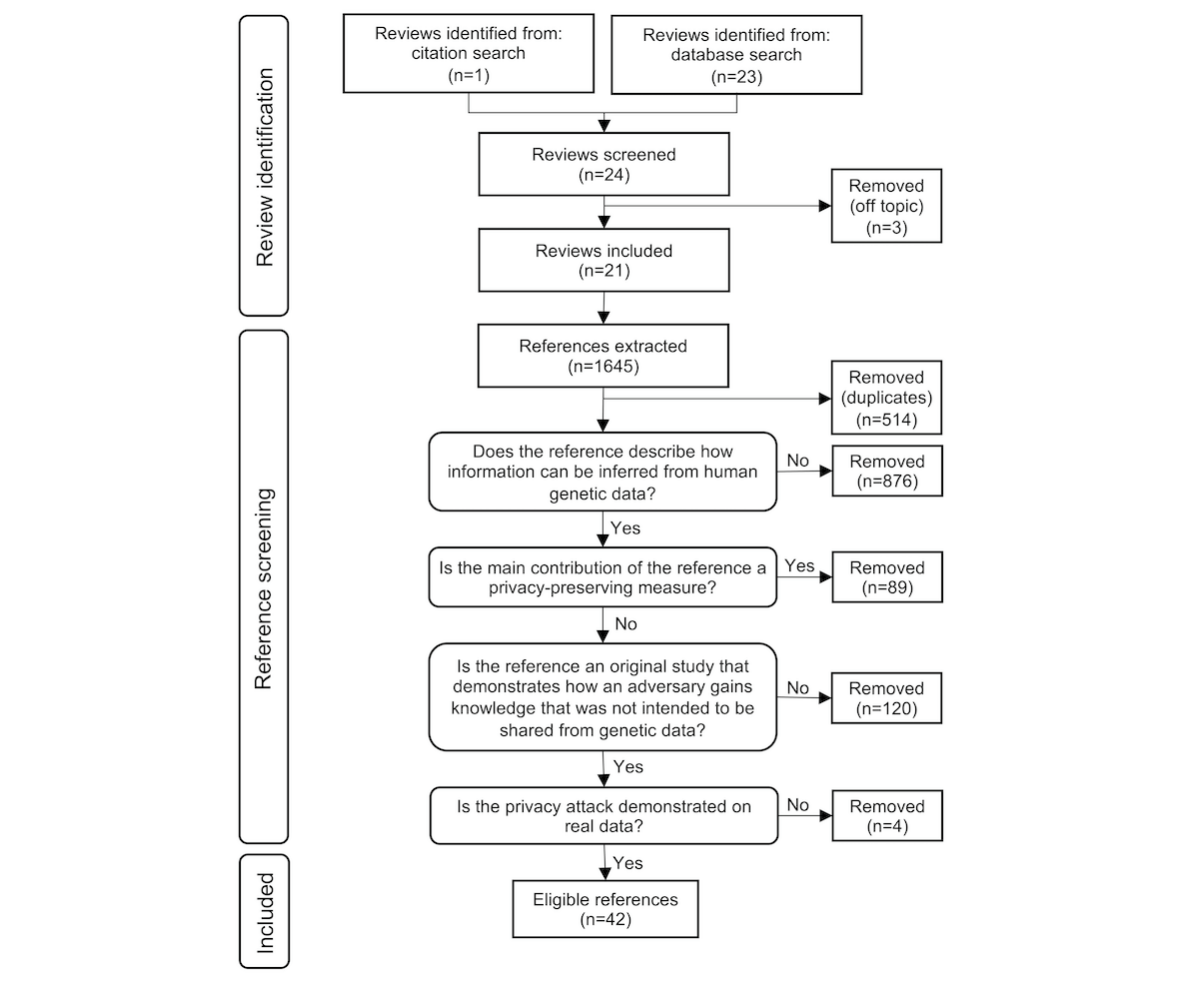
Flowchart overview of the 2-step literature review process: identification of relevant reviews, followed by extraction and screening of references.

To get a comprehensive overview of the types and aspects of genetic data sets that are vulnerable to reidentification attacks, as well as the methods, databases, and know-how used for these attacks, we searched for studies that demonstrate a privacy attack on genetic data. We did not aim to establish an exhaustive overview of all published privacy attacks but aimed to get a comprehensive understanding of the most vulnerable features of genetic data. Therefore, we first searched for recent reviews published on the topic of genomic privacy using ProQuest. Using the search terms (ti(*genom* OR *genetic*) AND ti(privacy OR re-identification OR reidentification OR “data security”)) and (pd(>20170101)) and (at.exact(“Review”)), we identified 23 reviews, of which 3 (13%) were discarded because they were off topic. One additional review was identified during the literature research and added to the selection (refer to Multimedia Appendix 1 [35-55] for an overview of the included and excluded reviews), resulting in a final sample of 21 reviews. In a second step, we extracted all references cited in the reviews (n=1645) and identified all original research studies that demonstrate a privacy attack on genetic data. After the removal of 514 duplicates and 876 reference studies that did not contain any description of information inference from human genetic data, we first excluded 89 studies whose main contribution was the presentation of privacy-preserving measures to exclude privacy attacks that were performed only for the purpose of proving the efficiency of the proposed counter methods. Next, we excluded 120 studies that did not present original research and were purely associative (ie, did not demonstrate how an adversary gains knowledge that was not intended to be shared from genetic data) as well as 4 studies that did not demonstrate the attack on real data. This process resulted in the selection of 42 unique studies (refer to Figure 1 for an overview of the process and Table S1 in Multimedia Appendix 1 for an overview of the eligible attack studies). Extending on the framework by Mohammed Yakubu and Chen [12] and Lu et al [56], we categorized attacks into (1) identity tracing (attacker triangulates the identity of an individual), (2) inference (attacker uses an individual’s genetic data to infer sensitive attributes such as disease or drug abuse or to infer additional data or cross-link records across databases), and (3) membership attacks (attacker uncovers membership of an individual in a data set). We evaluated the type and components of genetic data exploited for this attack as well as the effort and resources used for it (time, expertise, databases, and computation power) and its success rate if sufficient information was reported in the study. The initial evaluation was conducted by one reviewer and independently verified by another. Table S1 in Multimedia Appendix 1 presents a detailed overview of the attack studies.

## Results

### A Comprehensive Overview of Privacy Risks in Genetic Data Sets

**Figure 2:**
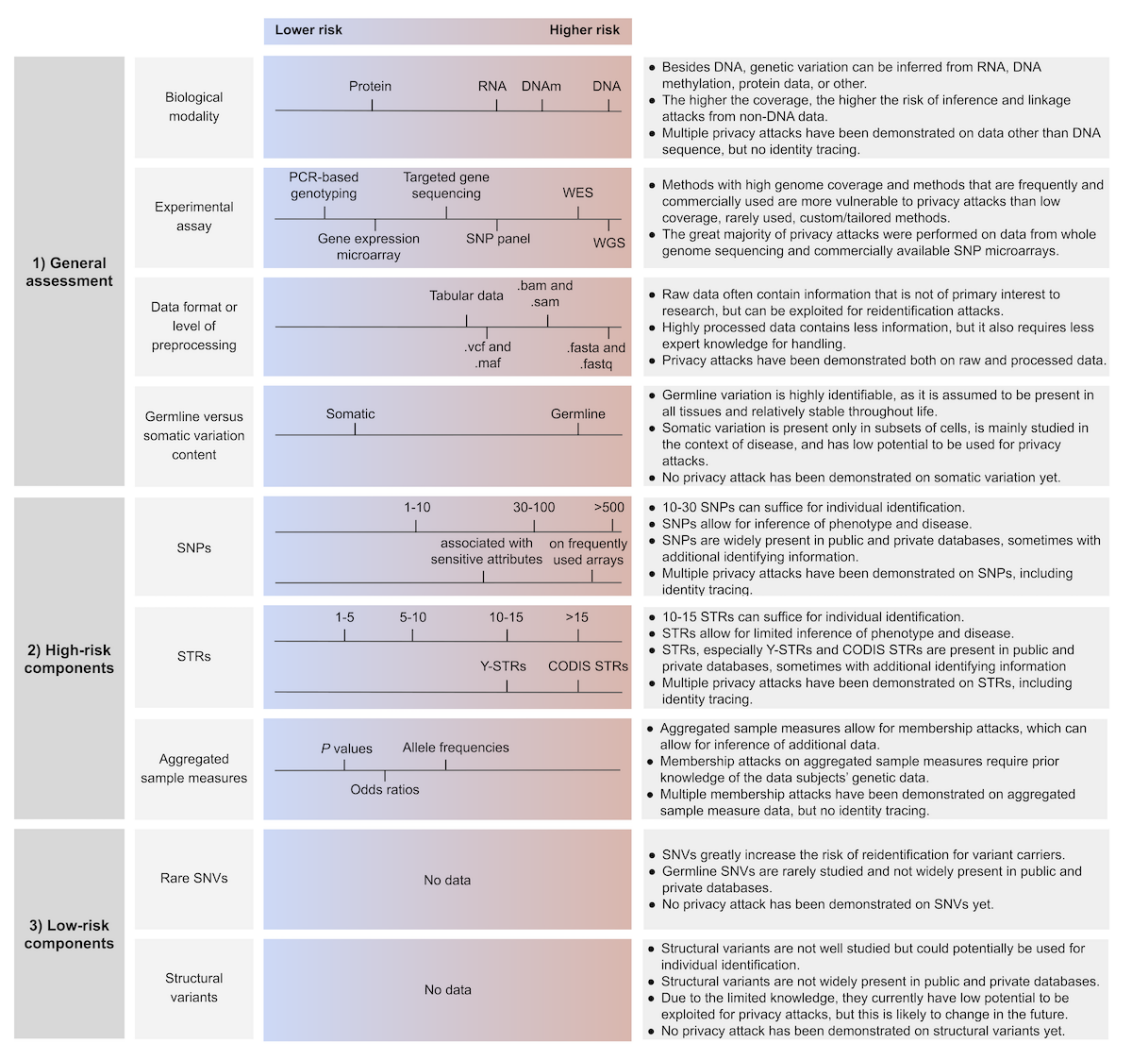
Overview of the privacy-critical features of genetic data sets, with exemplary values and key points to consider for risk assessment. CODIS: Combined DNA Index System; SNP: single nucleotide polymorphism; SNV: single nucleotide variant; STR: short tandem repeat; WES: whole exome sequencing; WGS: whole genome sequencing; Y-STR: short tandem repeat on the Y chromosome.

On the basis of our literature review, we created an overview of the parts and aspects of genetic data that are commonly exploited in privacy attacks and that should therefore be taken into consideration when performing a risk assessment on genetic data. The goal of this overview is to provide data processors, who may not be experts in genomic data privacy, with essential background knowledge about the privacy vulnerabilities associated with genetic data. This understanding will help them identify privacy-critical aspects and serve as a starting point for conducting risk assessments on genetic data sets. Notably, the reidentification risks associated with data that complement genetic data (eg, clinical data and demographic data) as well as aspects of the data environment (access and governance) are crucial for a comprehensive risk assessment [57], but these aspects are not in the scope of this research. From our literature review, we synthesized 9 features that are both inherent to any genetic data and informative about privacy risk (Figure 2). The features are not mutually exclusive. Instead, they represent different “views” on genetic data and highlight various aspects that should be considered in a privacy risk assessment. For each feature, we lay out why this feature is associated with privacy risk by summarizing the relevant evidence in the scientific literature, and we assess the criticality of these attacks. In addition, we provide guiding questions that help to assess the risk of a given data set. The features can be divided into three groups:

1. The first 4 features are general categorizations of the genomic data set and serve as a very rough estimate of the amount of privacy-critical information in the data.
2. The next 3 features are specific genomic features that are known to be a high risk for privacy. Their assessment is critical for estimating the reidentification risk.
3. The last 2 features are genomic features that have not been exploited for privacy attacks yet but should still be considered and could present a risk if they are present to a high degree in the data.

We summarize our findings in an overview figure, which lists the 9 features and their relevance for privacy. While it is challenging to define clear risk thresholds, there is a recognized need for practical guidance and orientation. To address this, we provide a scale that ranges from lower to higher risk and offer illustrative examples derived from the overview of privacy attack studies. These scales and examples serve as the initial guidance for risk assessment, emphasizing their purpose as guiding principles rather than exact measurements. The assessment of each individual feature is intricate and thoroughly explained in the corresponding sections. In addition, while the scales offer a framework to compare and assess different features, it is crucial to consider all features comprehensively to arrive at a conclusive assessment. Furthermore, the text sections highlight important interactions that arise from the comprehensive evaluation of these features.

Table S1 in Multimedia Appendix 1 presents a detailed description of the original attack studies.

### Evidence of Privacy Risks in Genetic Data

#### Part 1. General Assessment

##### Biological Modality

While most privacy attacks have been demonstrated on DNA sequence data, other types of molecular data (eg, DNA methylation data or data derived from RNA) are also considered genetic data under General Data Protection Regulation, can also be identifiable, and have also been exploited for attacks [58-67]. Attacks on these types of data are performed mainly by 3 mechanisms. The first mechanism is direct extraction of DNA sequence from raw or low-processed data. This is possible, because even if not of primary interest, DNA sequence information is often a by-product of gene expression or DNA methylation studies [68-70]. For example, Gürsoy et al [70] demonstrated how genetic variants can be called from raw RNA sequencing data. The second mechanism is inference of DNA sequence, for example, through known associations of genetic sequence and gene expression or other modalities. For example, Schadt et al [65] used gene expression data of individuals (40,000 transcript counts) to infer genetic variants (1000 SNPs), which allowed them to determine with high certainty whether individuals with known SNPs were members of a gene expression study cohort (N=378). They also assessed the success rate of matching gene expression records to SNP records in a simulated cohort of 300 million individuals and correctly matched 97.1% of the records, demonstrating the feasibility of cross-linking these data types, which since then has been confirmed in additional studies [60,62,63]. Less literature has been published on other types of data, such as protein or epigenetic data (eg, DNA methylation), but similar proof of concept of cross-linkage to SNP data has been demonstrated in prior studies [58-60,63,64,66,67,71]. In the third mechanism, sensitive information such as disease phenotypes, demographic information, and behavioral traits is inferred from gene expression, protein levels, or other modalities (eg, age [72], cigarette smoking, and alcohol consumption [59] from DNA methylation).

However, such inference and linkage are not error free. For example, in the study by Schadt et al [65], the accuracy of the imputed SNPs from gene expression data was low (average Pearson correlation coefficient was 0.35 between true and inferred genotype). It is not clear whether such imputed data could be used for privacy attacks in the real world, such as in an identity tracing attack (eg, via upload of the imputed genetic data to GEDmatch or other). Considering that previous successful identity tracing attacks have used >500,000 SNPs [6], the inference of 1000 SNPs (with errors) may not be sufficient for such an attack. If the reconstruction of a larger set of SNPs were attempted, it is likely that the initial imputation error would propagate and thereby reduce the probability of a successful identity tracing attack. Furthermore, Schadt et al [65] reported much lower matching performance if training and test data stem from different array manufacturers, a scenario that is likely to occur in real-world data. Finally, although biological associations between genomic variants and gene expression are publicly accessible, substantial expert knowledge is still required for accessing this information and implementing the attack. Similar limitations apply to all the aforementioned studies. Altogether, data sets of RNA, protein, or epigenetic data, especially if they are large (eg, genome-wide), do allow for linkage and inference attacks. However, true reidentification would require matching the inferred genetic or phenotypic information to databases with identifying or quasi-identifying information in a next step, and no such full identity tracing attack starting with data other than DNA sequence has been demonstrated yet.

The guiding questions in this context are as follows:

- Do the data contain DNA sequence information directly (eg, DNA sequencing reads)? If yes, could the data be processed such that sequence information is no longer available (eg, report DNA methylation levels in percentage instead of providing raw sequencing read files)?
- Could DNA sequence information be inferred from the data (eg, via biological correlations such as expression or methylation quantitative trait loci)?
- What sensitive information could be inferred from the data (eg, age, sex, diseases, or physical traits)?

##### Experimental Assay

Knowing the experimental assay that was used to generate the data can already provide a first estimate of its information content and linkability. For example, sequencing-based assays generally produce very rich data (eg, high genome coverage and high precision, such as whole genome DNA sequencing), whereas polymerase chain reaction–based genotyping assays provide more sparse data (eg, information on only 1 nucleotide of the DNA sequence). However, genome coverage alone (ie, the percentage of all base pairs or loci of the genome covered by the method) is not a reliable proxy for privacy risk. In some circumstances, a data set with only 10 sequenced positions of the DNA could in fact be more critical than a data set containing hundreds of positions, if those 10 positions are in highly identifiable loci. However, as a very rough indicator of information content, we believe it is still valuable to consider the genome coverage of the data as one of many factors in the risk assessment. In many cases, the rule of thumb that more sequence information equals higher information content and hence risk of cross-linking, inference, and reidentification is true. Nevertheless, these aspects need to be carefully evaluated together with the biological modality of the data, the level of processing, and the specific content of the data.

It is also important to consider that data produced with frequently used methods, such as commercially available kits (eg, SNP microarrays), often target the same genetic variants that are also interrogated by DTC-GT companies and genome-wide disease association studies and can thus more easily be linked to public data and exploited for privacy attacks than data generated with tailor-made, targeted analysis methods (refer to the study by Lu et al [73] for an overview of genotyping arrays commonly used by direct-to-consumer companies). Finally, as nearby variants are more likely to be correlated, it is also important to consider how the genetic information in the data is spread across the genome. A targeted assay that reads all SNPs within a specific gene likely carries less information than an assay that interrogates the same number of SNPs distributed across the full genome, as nearby SNPs are more likely to be correlated [74]. In line with these arguments, the great majority of published privacy attacks were performed on data obtained from whole genome sequencing and commercially available SNP microarrays (ie, rich, genome-wide data in the order of hundreds of thousands of SNP loci from a commercial assay).

The guiding questions in this context are as follows:

- Which method was used to generate the data? Does this method produce rich or sparse data? (What percentage of all base pairs or loci of the genome are covered by the method?)
- How do the data produced with this method cover the genome (ie, genome-wide vs targeted approach)?
- How likely is it that data generated with the same method are present in publicly available databases (ie, commercial assay vs custom)?

##### Data Format or Level of Processing

The format of the data gives some indication on its processing level and can thus help to estimate its information content. Genetic data processing consists of cleaning, filtering, normalizing, and reducing raw data to a version that contains only the information that is relevant for its intended use. Important standard formats in genomic sequencing experiments sorted from raw to processed are *.fasta* and *.fastq* (raw nucleobase reads); *.bed*, *.bam*, and *.sam* (reads aligned to reference genome); *.vcf* and *.maf* files (deviations from the reference genome only), whereas highly processed data are often represented in tabular (.*csv* and .*tsv*) or otherwise structured form (.*json*, .*xml*, or other) containing only variants or regions of interest. Raw or low-processed data (.*fasta*, .*fastq*, .*bed*, .*bam*, or .*sam*) often contain information that is not of primary interest to research but can be exploited for reidentification attacks (eg, raw read files from gene expression studies can contain genomic variant information [63]). While the possibilities for privacy attacks are greater in raw data, it is important to note that the required effort and expert knowledge for handling these data are usually higher than those for processed data, where genetic variants such as SNPs do not need to be extracted.

The guiding question in this context is as follows:

- If the data are in a raw or semiprocessed format, do the data contain any information that is not directly relevant for their intended use?

##### Germline Versus Somatic Variation Content

Genetic variants found in an individual’s genome can be categorized into germline and somatic variants. This categorization is specific to individuals and depends on the heritability of the variant (ergo, its presence in the individual’s reproductive tissues). Heritable variants are categorized as germline (ie, present in germ and usually also in somatic cells) and nonheritable variants are categorized as somatic (ie, present in somatic cells only). In the context of genetic privacy, it is important to understand that germline variation comprises all variants that can be assumed to be present in every cell of the body, are not expected to change much throughout the lifetime of an individual, are inherited from parental DNA, and are expected to be passed to the offspring. Such variation can inform about identity, ancestry, and kinship and is, therefore, used by DTC-GT providers, forensics, and genetic genealogy services. The most prominent example for germline variation are SNPs, as variation found at known SNP loci is generally assumed to be germline. (However, the terms germline variants and SNPs cannot be used interchangeably, as they refer to different concepts: germline describes the heritability, and SNP describes the type of variant and its frequency in the population.) Overall, germline variants are not only highly relevant for individual identification because of their stability and omnipresence across tissues but are also of great interest for scientific research. Associations of germline variants to disease, physical traits, or other biomedical modalities are well studied, with results being publicly accessible. As such, germline variants are vulnerable to identity, inference, and linkage attacks, and indeed, all the reviewed privacy attacks targeted germline variants.

In contrast, somatic variants are acquired during life (after fertilization) and are usually present only in specific, nonreproductive tissues or even only in single cells or cell populations. They are intensively studied in the context of diseases (eg, cancer), and as they are often found to be associated with diseases, data on somatic variants could be used to infer sensitive attributes about data subjects. However, their low association with identity and use limited to clinical diagnostics and scientific research makes it very difficult to cross-link them to databases with identifying or quasi-identifying information. DTC-GT companies, forensics services, or genetic genealogy services do not use somatic variants to determine identity, familial relations, or ancestry, as somatic variation is neither stable nor present in all tissues and cells (usually found only in a fraction of cells analyzed in a sample). A linkage attack based on somatic variation would require a matching data record of the same tissue, ideally taken at a similar time in life, which is unlikely to exist for most cases (as somatic variant patterns can change rapidly, eg, in cancer tissue). No identity tracing, inference, or membership attack based on somatic variation data has been published yet, and considering its low potential for identifiability, somatic variation data can currently be considered a low risk for reidentification attacks.

To determine whether a variant is germline or somatic, one would ideally analyze multiple samples from one individual to determine whether the variant is present in germ cells or only in specific somatic cells. In practice, experts can assess the status of a variant from its sequencing read signal (determining whether it is present in all cells of the sample or only in a few), genomic location, and type alone by comparing it to public knowledge of known loci of germline and somatic variation or through computational approaches [75]. In processed genetic data, variants which are with high certainty germline have often already been identified and are indicated as such (eg, SNPs are identified by a specific reference SNP cluster ID, such as “rs343543”), whereas somatic variants are described by standard mutation nomenclature (eg, single nucleotide variants [SNVs] are described by the Human Genome Variation Society nomenclature, containing the reference genome used; the genomic location of the variant; the nucleotide in the reference sequence; and the detected nucleotide, such as “NC_000023.9:g.32317682G>A”). Furthermore, the type of tissue that was used to generate genetic data, most importantly whether samples were taken from healthy or tumor tissue, can also give some indication on the amount of germline variation included in the data. When analyzing tumor tissue data, germline variations such as SNPs are typically removed during processing, as the focus is on studying somatic variation. However, especially if the data are raw and unfiltered, they often contain germline variants irrespective of whether they were taken from healthy or tumor tissue and must hence be considered a higher risk for reidentification. Therefore, while data that are both derived from tumor tissues and highly processed are often a low privacy risk, the amount of information on germline variation that is contained in the data needs to be assessed case by case. Public databases (eg, dbSNP, hosted by the National Institutes of Health’s National Center for Biotechnology Information) store information about the genomic locations and population frequencies of SNPs and can be used to search data for this important type of germline variation.

The guiding questions in this context are as follows:

- Was germline or somatic variation of primary interest when generating or processing the data?
- If somatic variation was of primary interest, was germline variation removed from the data?

#### Part 2. High-Risk Components

##### SNPs

SNPs are germline SNVs that are present in >1% of the population. They are highly relevant features for individual reidentification and the most privacy-critical component of genetic data sets. Because SNPs usually have 2 different states (ie, a common or reference and a rare nucleotide) and human somatic cells have 2 DNA copies (ie, are diploid), an individual usually has 1 of 3 different states at a SNP locus, often represented as 0,1, and 2 (0 represents 2 copies of the common variant [ie, homozygous for major allele], 1 represents 1 copy of the common variant and 1 copy of the rare variant [heterozygous], and 2 represents 2 copies of the rare variant [homozygous for minor allele]). Knowing an individual’s state at 30 to 80 statistically independent SNPs (or a random set of approximately 300 SNPs) can suffice for individual identification [76-79], yet commonly used SNP or genome sequencing assays often read hundreds of thousands of SNPs at once. As germline variation, SNPs are assumed to be stable and present in every cell of the body, signifying that they can identify individuals across samples taken at different times or from different tissues. As they are heritable, DTC-GT providers and forensic institutes compare SNP patterns of individuals to determine familial relations and ancestry [80]. Furthermore, SNPs are associated with physiological traits (eg, skin, hair and eye color [2,3], facial features [81], BMI [82], and height [4]), ethnicity [1], and susceptibility to diseases [5], making them central to research and genetic testing (refer to the study by Dabas et al [83] for a review of association of SNPs with externally visible characteristics).

SNP data can be directly used for reidentification by matching it to publicly accessible databases, as demonstrated in the reidentification attack by Erlich et al [6], who uploaded SNP data (700,000 SNPs) from an anonymous study participant to the genetic genealogy website GEDmatch and identified the participant’s surname through matches with relatives. Such identity tracing attacks are possible because millions of people send their DNA to DTC-GT companies such as AncestryDNA, 23andMe, FamilyTreeDNA, or MyHeritage [84], and many also decide to share their genetic data on publicly accessible websites, such as GEDmatch, the Personal Genome Project [85], or OpenSNP [86]. Enabling individuals to identify and contact relatives, learn about their ancestry, disease predispositions, and contribute their data to research, these platforms often contain genetic data accompanied by information about an individual’s diseases and traits or even personal data such as place of residence, age, sex, surname, or phone number. In addition, there is a wealth of publicly accessible knowledge on associations of SNPs with physical features, diseases, other genetic variants or genetic modalities (eg, gene expression and DNA methylation; eg, dbSNP database [87], the GWAS catalog [5], the International Genome Sample Resource from the 1000 Genomes Project [88], and data from the HapMap project [89]), which can and have been exploited for completion and inference attacks (eg, inference of additional genetic variation in genomic regions that were not studied originally, other biomedical modalities such as gene expression and DNA methylation, or physical attributes [9,90-96]). For example, Humbert et al [92] predicted phenotypic traits (eye, hair and skin color, blood type, and more) of individuals from their SNP data (20 SNPs) using publicly available knowledge on SNP-phenotype associations from the public database SNPedia and used this information to cross-link individuals between genetic and phenotypic data sets. In addition, Humbert et al [92] inferred additional and sensitive information (eg, susceptibility to Alzheimer disease) from the SNP data. However, this linkage attack had a success rate of only 5% (ie, proportion of correctly matched individuals) in a data set of 80 individuals and is likely to perform worse in more realistic scenarios with larger data sets. Nyholt et al [9] imputed the status of multiple risk variants for Alzheimer disease in the published genome of Dr James Watson [94] from SNPs in nearby genomic regions, although the respective gene had been masked. Edge et al [90] cross-linked individuals in SNP and short tandem repeat (STR) data sets, a highly identifiable type of genetic variation that is used in forensics, by imputing STR from SNP data (642,563 loci). In a highly debated study, Lippert et al [93] developed a model to predict phenotypic traits (facial structure, voice, eye color, skin color, age, sex, height, and BMI) from whole genome sequencing (WGS) data containing >6 million SNPs and used it to cross-link high-resolution face photographs of individuals to their genetic data in a cohort of 1061 study participants. In a real-life scenario, photos and personal data from social media could be exploited for such an attack and matched to the inferred phenotype. However, it has been argued that the predictive power in this study stems mainly from the estimation of the participant’s ancestry and sex [97] and that the attack is unlikely to be successful in the real world and with more realistic, lower-quality images [98]. Furthermore, large, genome-wide association studies indicate that the currently known associations between SNPs and facial structure, voice, height, and BMI are too small to be useful for accurate phenotype prediction on an individual level; however, this will likely improve in the future. Nevertheless, other characteristics, such as ancestry, eye, hair color, and skin color, can be inferred from specific SNPs with high accuracy, and corresponding DNA phenotyping kits are already commercially available and used in forensics today [99]. As a small number of SNPs can already uniquely identify an individual and SNPs are widely available in public databases together with identifying and quasi-identifying information, SNPs must be considered a high risk for privacy and data sanitization efforts (eg, as proposed by Emani et al [100]) should be used in any genetic data set containing >20 SNPs.

The guiding questions in this context are as follows:

- How many SNPs do the data contain (directly or indirectly)?
- Are the SNPs in close proximity or spread across the genome (nearby SNPs are more likely to be correlated and thus often contain less information than statistically independent SNPs)?
- Are the interrogated SNPs frequently assessed in research or by DTC-GT providers (ie, how likely is it that they can be linked to publicly available, identifying data sets)? The study by Lu et al [73] presents an overview of genotyping arrays commonly used by direct-to-consumer companies.
- Are all SNPs relevant to the intended use of the data or could some be removed from the data?
- What sensitive information could be inferred from the data (eg, diseases and physical traits)?
- Could additional DNA sequence information be inferred from the data (eg, association with STRs or other)?

##### STRs

The human genome contains more than half a million regions of repetitive units of 2 to 6 bases, the so-called STRs or microsatellites [101]. The number of repeats in these regions is highly variable across individuals and can affect protein function or expression or be linked to medical conditions or physical traits [102]. Knowing the repeat numbers of as little as 10 to 30 STRs can suffice for individual identification. Because of their high identifiability, STRs are used to determine identity and kinship in forensics, law enforcement, paternity testing, and genetic genealogy. For example, the Combined DNA Index System (CODIS; a set of 20 STRs) is used to connect suspects to crime scenes or establish identity of missing persons. While CODIS STRs are usually not of interest in research studies or genetic genealogy, STRs on the Y chromosome (ie, Y-STRs, only present in male individuals) are included in several DTC-GT kits, where they are used to identify relatives along the paternal ancestry line (eg, “Y-STR Testing” by FamilyTreeDNA). Consequently, several large databases of STR loci with accompanying identifying and quasi-identifying information exist (eg, mitoYDNA from mitoYDNA Ltd). In addition, the CODIS forensic database and analysis software contains genetic data and identifying information from >14 million individuals in the United States alone [103].

Several studies demonstrate reidentification attacks on Y-STRs. Gitschier et al [104] provided first evidence for surname inference from Y-STRs by matching genetic STR profiles of anonymous study participants from the international HapMap project [89] to 2 genetic genealogy databases (Ysearch and Sorenson Molecular Genealogy Foundation [SGMF]). Later, Gymrek et al [105] demonstrated that it is not only possible to infer surnames from STR data (eg, 34 Y-STR loci extracted from WGS data) but also to triangulate the actual identity of data subjects with high probability using publicly accessible genealogy databases, record search engines, obituaries, and genealogical websites. The authors attempted this for 10 study participants of the 1000 Genomes Project and correctly identified 5 out of 10 individuals. It is important to note that STR data can also be fortuitously included in genetic data derived from targeted gene or WGS, even if they are not of primary interest for the study. Moreover, STR markers can be imputed from genetic data sets that do not even cover STR regions by exploiting known associations between SNPs and STRs [90]. While the authors of this study report a low imputation accuracy for STRs from SNPs (likely too low to reliably impute full STR profiles even from large SNP data), they did demonstrate the ability to cross-link records across SNP and STR databases. In detail, they correctly matched 90% to 98% of paired SNP (642,563 loci) and STR data records (13 STRs) to each other, and such successful linkage has also been demonstrated elsewhere [106].

Due to the high association of STRs with identity, any genetic data that directly (eg, repeat numbers for specific STR regions) or indirectly (eg, WGS data covering STR regions) contain >10 STR regions could be considered identifiable. However, the actual risk of reidentification depends on the availability of STR databases with identifying and quasi-identifying information and the ability to cross-link records. It is important to note that the databases used in the seminal study by Gymrek et al [105] (ie, Ysearch and SGMF) are no longer available (Ysearch, belonging to FamilyTreeDNA, closed in 2018, and SGMF, belonging to Ancestry, was shut down in 2015), and access to the CODIS database is restricted to criminal justice agencies for law enforcement identification purposes. However, databases from DTC-GT providers (eg, FamilyTreeDNA) and public platforms (eg, mitoYDNA) are still available and allow uploading results from third-party providers; therefore, an attacker could fabricate a genetic testing result from STR data [107,108] and reproduce the demonstrated surname inference attacks. From information about possible surnames, sex, and residence inferred from matches on the platform, the triangulation of identity could be possible with the help of additional publicly available resources [105,109]. However, such an attack would only be possible on male data records (ie, Y chromosome based) and is not guaranteed to find matches that allow surname inference; the success rate in the demonstrated attack was 11.9% (109/911 cases), and the 2 previous studies used >30 STR loci (all located in close vicinity of each other and on the Y chromosome). Furthermore, the know-how and effort necessary for such an attack is high. Finally, even if genetic matches or surnames are identified, the reconstruction of identity from surname is not trivial and can take months to complete, as others have pointed out [110]. Still, because of their high identifiability potential and their use in DTC-GT, paternity testing, and forensics, STRs should be removed from genetic data if they are not of primary interest and otherwise considered a high risk for privacy.

The guiding questions in this context are as follows:

- Do the data directly or indirectly (eg, STRs in raw data and STRs imputable from SNPs) contain >10 STR loci?
- Are these STR loci either (1) part of the CODIS system or (2) on the Y chromosome (ie, high linkability)?
- Could additional DNA sequence information be inferred from the data (eg, association with SNPs or other)?

##### Aggregated Sample Measures

Aggregated sample measures, that is, variables that are the result of aggregating genetic data across multiple samples can also be exploited for privacy attacks (reviewed by Craig et al [111]). The most prominent examples are summary statistics from association studies such as SNP frequencies, odds ratios, or correlation coefficients. However, the limited information content in these summary statistics usually only allows for membership attacks, that is, assessing whether an individual of known genetic background is part of a study group or database or not [112-114]. Multiple studies demonstrate such an attack [113,115-119], although Homer et al [114] were the first to explain how membership of an individual in a mixture can be predicted from the reported SNP allele frequencies (ie, if SNPs of that individual are known, in this case >10,000 SNPs). The authors accomplished this by comparing the reported study allele frequencies to allele frequencies in a reference cohort of similar ancestry (obtained from public resources) and detecting the bias introduced by the sample of interest. Their method performed well even if the individual’s contribution to the mixture was <1%, and this method can easily be extended to predicting membership from aggregated data from a study cohort. In response to that, the US National Institutes of Health has restricted the publication of aggregate GWAS results in their databases [120]; however, the feasibility of the attack has been critically discussed. Its power depends on the size and quality of the actual and reference cohorts, the number of reported SNP allele frequencies, prior knowledge of the attacker, and the fulfillment of several underlying assumptions, many of which are likely not fulfilled in practice [115,116,121,122]. Aside from membership attacks, it was also shown that aggregate results, such as linear models that have been fitted to study data or polygenic risk scores, can be exploited to predict sensitive attributes and genotypes via model inversion [28,123]. However, this attack required background information on the data subject and on the distribution of variables in the study data. Furthermore, its performance is limited by the predictive power and complexity of the fitted model. Membership and attribute inference attacks on aggregate data can reveal demographic, genetic, and phenotypic information (such as country or place of residence due to participation in a local study, ethnicity, disease, age group, or presence of specific genetic variants due to descriptions of inclusion or exclusion criteria in the cohort) and can thus facilitate linkage and identity tracing attacks, which is why they can be a risk for privacy. However, no identity tracing attack based on aggregate data has been demonstrated yet.

The guiding question in this context is as follows:

- What sensitive information could an attacker gain from ascertaining the membership of an individual to the data set (eg, geographic information, sex, disease, and age)?

#### Part 3. Low-Risk Components

No privacy attack has been demonstrated on these components, but due to their high association with identifying and sensitive attributes, we recommend including them in the risk assessment.

##### Rare SNVs

Rare SNVs are single nucleotide substitutions that are present in <1% of the population. They may be somatic or germline and can be associated with pathological conditions and thus reveal sensitive information. Furthermore, while less informative than common SNVs (ie, SNPs) from an information theoretical standpoint, rare variants greatly increase the risk of reidentification for the small subpopulation of variant carriers. However, because of their low frequency in the population, germline SNVs are rarely the target of large scientific studies (eg, for phenotype or disease association) and have very limited use for ancestry and disease susceptibility analysis. Therefore, most DTC-GT providers and research studies specifically target regions of common genetic variation (eg, SNPs) and either use assays that do not detect SNVs or remove them during preprocessing, making it very unlikely that a set of SNVs could be linked to any database with quasi-identifying information. No identity tracing, completion, or inference attack has been published on SNVs yet; therefore, they can currently be viewed as a low risk for reidentification, despite their high theoretical potential for identifiability.

The guiding questions in this context are as follows:

- What sensitive information could be inferred from the data (eg, diseases and physical traits)?
- Could additional DNA sequence information be inferred from the data (eg, association with SNPs or other)?
- Are there any databases that could be used to cross-link the data to identifiable data, and how accessible are the databases?

##### Structural Variants

The study of structural variants (SVs) in the human genome is in its early stages, but it is already clear that it accounts for even more individual variation than SNPs [124,125]. The best-studied type of SVs is copy number variation (CNV), that is, deletions and duplications of regions larger than 50 base pairs. CNVs can be used as measures of relatedness and identifiers of population origin [126], have a strong impact on gene expression [127], and could allow for the inference of physical features [128] and pathological conditions [129], thereby revealing sensitive information of data subjects. However, CNVs are still not well studied, and sequencing technologies have only recently progressed to a level that allows to capture their full scope in the human genome (reviewed by Mahmoud et al [124]). Most importantly, human CNV databases are very scarce in comparison to databases of SNVs (refer to the study by Ho et al [130] for an overview of the available human SV reference sets), and they are currently not used for genetic genealogy analyses, making it difficult to link CNVs across databases to obtain identifying information. A privacy attack based on CNVs or any other type of SV yet remains to be demonstrated. Finally, it is important to note that many SVs that are assessed in medical and research studies are somatic, that is, nonhereditary, not present in all cells of the body, not stable, and thus not strongly associated with identity. For example, tumor tissue is characterized by frequent and dynamic changes in SVs (eg, CNVs in tumor tissue, also referred to as CNAs), which are likely neither directly nor indirectly identifiable. Therefore, the risk of reidentification from SVs can currently be considered low, but the growth of public databases and their use in genealogical or clinical research should be monitored. The same holds true for common SVs, such as CNVs that occur in >1% of the population and are hence classified as polymorphisms (ie, CNPs). Little is known about the population frequencies of CNVs, and while public databases are growing, no privacy attack based on CNPs has been demonstrated yet. Due to the limited knowledge about CNPs or other common SVs in the population, their presence in genetic data is difficult to assess, and they can be considered a low risk for reidentification at the current time.

The guiding questions in this context are as follows:

- What sensitive information could be inferred from the data (eg, diseases and physical traits)?
- Could additional DNA sequence information be inferred from the data (eg, association with SNPs or other)?
- Are there any databases that could be used to cross-link the data to identifiable data, and how accessible are the databases?

## Discussion

### Limitations

It is important to acknowledge some key limitations of our review. First, it is possible that we may have missed relevant studies. This is particularly true for recent research, as our search was confined to original studies referenced in existing reviews. While the search strategy was designed to retrieve the most pertinent studies, it carries the risk of overlooking lesser-known or very recent studies. Therefore, we recommend conducting periodic reviews to stay updated with scientific advancements and changes in the availability of public genetic data that may contain (indirectly) identifying information susceptible to identity tracing attacks. Second, even under the assumption that all relevant literature was considered, it is still possible that we may have overlooked certain vulnerabilities. This is known as the “proof of nonexistence fallacy”—the absence of evidence for risk does not imply the absence of those risks. Finally, it was necessary to balance our aim of providing a comprehensive and evidence-based overview of genetic privacy vulnerabilities with our aim of providing practical and useful guidance. Therefore, we provide both a detailed assessment (refer to the *Results* section and Table S1 in Multimedia Appendix 1) as well as a simplified overview (Figure 2). However, this trade-off necessitated compromises in practical utility on one hand and scientific exhaustiveness on the other hand.

### Conclusions

On the basis of the findings of this review, it can be argued that the privacy risks of genetic data vary greatly between data sets. Considering all genetic data at all times as information relating to an identifiable natural person is not correct, and it is becoming apparent that reidentification risk in genetic data must be assessed on a case-by-case basis and under the consideration of all the means reasonably likely to be used [131]. However, while efforts are underway [132], no practical guidelines or recommendations for performing such a reidentification risk assessment on genetic data have been proposed yet. On the basis of a review of the scientific literature on privacy attacks on genetic data, we provide an overview of genetic data privacy risks that can guide data processors in risk assessment by providing the necessary background knowledge and an overview of the existing evidence. We believe that a careful examination of the 9 described features in the data set at hand (biological modality or type of data, experimental assay, data format or level of processing, germline vs somatic variation content, content of SNPs, STRs, aggregated sample measures, rare SNVs, and SVs) provides a strong foundation for a data risk assessment. While completely eliminating the possibility of reidentification is rarely achievable, a more practical approach of risk minimization is warranted [133,134], accompanied by organizational and technical measures to safeguard genetic data from reidentification attack attempts and a transparent communication of the remaining risks to data subjects.

## Appendix

Multimedia Appendix 1 List of identified reviews and a table with the description and evaluation of original privacy attack studies.

Multimedia Appendix 2 PRISMA checklist.

## Abbreviations

CNV: copy number variation
CODIS: Combined DNA Index System
DTC-GT: direct-to-consumer genetic testing
SGMF: Sorenson Molecular Genealogy Foundation
SNP: single nucleotide polymorphism
SNV: single nucleotide variant
STR: short tandem repeat
SV: structural variant
Y-STR: short tandem repeat on the Y chromosome
