# Supplementary material for: Assessing Privacy Vulnerabilities in Genetic Data Sets: Scoping Review
Source: JMIR Bioinform Biotechnol. 2024 May 27;5:e54332. doi: 10.2196/54332 (PMC11165293; doi:10.2196/54332)
Supplement: Multimedia Appendix 1 [file bioinform_v5i1e54332_app1.docx]

# Supplementary Information

List of reviews included in the literature review. Reviews were identified by ProQuest search (n=20), except indicated otherwise (*) (n=1).

Abinaya, B., & Santhi, S. (2021). A survey on genomic data by privacy-preserving techniques perspective. Computational Biology and Chemistry, 93, 107538.

Azencott, C. A. (2018). Machine learning and genomics: precision medicine versus patient privacy. Philosophical Transactions of the Royal Society A: Mathematical, Physical and Engineering Sciences, 376(2128), 20170350.

*Ayday, E., & Humbert, M. (2017). Inference attacks against kin genomic privacy. IEEE Security & Privacy, 15(5), 29-37.

Aziz, M. M. A., Sadat, M. N., Alhadidi, D., Wang, S., Jiang, X., Brown, C. L., & Mohammed, N. (2019). Privacy-preserving techniques of genomic data—a survey. Briefings in bioinformatics, 20(3), 887-895.

Berger, B., & Cho, H. (2019). Emerging technologies towards enhancing privacy in genomic data sharing. Genome biology, 20(1), 1-3.

Bonomi, L., Huang, Y., & Ohno-Machado, L. (2020). Privacy challenges and research opportunities for genomic data sharing. Nature genetics, 52(7), 646-654.

Carter, A. B. (2019). Considerations for genomic data privacy and security when working in the cloud. The Journal of Molecular Diagnostics, 21(4), 542-552.

Clayton, E. W., Halverson, C. M., Sathe, N. A., & Malin, B. A. (2018). A systematic literature review of individuals’ perspectives on privacy and genetic information in the United States. PloS one, 13(10), e0204417.

Gürsoy, G. (2022). Genome Privacy and Trust. Annual Review of Biomedical Data Science, 5, 163-181.

Gürsoy, G., Li, T., Liu, S., Ni, E., Brannon, C. M., & Gerstein, M. B. (2022). Functional genomics data: privacy risk assessment and technological mitigation. Nature Reviews Genetics, 23(4), 245-258.

Knoppers, B. M., & Beauvais, M. J. (2021). Three decades of genetic privacy: a metaphoric journey. Human Molecular Genetics, 30(R2), R156-R160.

Lu, D., Zhang, Y., Zhang, L., Wang, H., Weng, W., Li, L., & Cai, H. (2021). Methods of privacy-preserving genomic sequencing data alignments. Briefings in Bioinformatics, 22(6).

May, T. (2018). Sociogenetic risks–ancestry DNA testing, third-party identity, and protection of privacy. New England Journal of Medicine, 379(5), 410-412.

Mohammed Yakubu, A., & Chen, Y. P. P. (2020). Ensuring privacy and security of genomic data and functionalities. Briefings in bioinformatics, 21(2), 511-526.

Oestreich, M., Chen, D., Schultze, J. L., Fritz, M., & Becker, M. (2021). Privacy considerations for sharing genomics data. EXCLI journal, 20, 1243.

Schwab, A. P., Luu, H. S., Wang, J., & Park, J. Y. (2018). Genomic privacy. Clinical chemistry, 64(12), 1696-1703.

Shen, H., & Ma, J. (2017). Privacy challenges of genomic big data. Healthcare and Big Data Management, 139-148.

Shi, X., & Wu, X. (2017). An overview of human genetic privacy. Annals of the New York Academy of Sciences, 1387(1), 61-72.

Stiles, D., & Appelbaum, P. S. (2019). Cases in precision medicine: concerns about privacy and discrimination after genomic sequencing. Annals of internal medicine, 170(10), 717-721.

Wan, Z., Hazel, J. W., Clayton, E. W., Vorobeychik, Y., Kantarcioglu, M., & Malin, B. A. (2022). Sociotechnical safeguards for genomic data privacy. Nature Reviews Genetics, 23(7), 429-445.

Wang, S., Jiang, X., Singh, S., Marmor, R., Bonomi, L., Fox, D., ... & Ohno‐Machado, L. (2017). Genome privacy: challenges, technical approaches to mitigate risk, and ethical considerations in the United States. Annals of the New York Academy of Sciences, 1387(1), 73-83.

List of reviews that were identified by ProQuest search, but not included in the literature review (off-topic).

Belani, S., Tiarks, G. C., Mookerjee, N., & Rajput, V. (2021). “I agree to disagree”: comparative ethical and legal analysis of big data and genomics for privacy, consent, and ownership. Cureus, 13(10).

Du, L., & Wang, M. (2020). Genetic privacy and data protection: a review of Chinese direct-to-consumer genetic test services. Frontiers in genetics, 11, 416.

Dugan, T., & Zou, X. (2017). Privacy-preserving evaluation techniques and their application in genetic tests. Smart Health, 1, 2-17.

Table S1: Table of attack studies with reference, attack type, description of the dataset under attack, information gained by the attack, requirements for success and the public resources used to implement the attack.

Abbreviations: AF = Allele frequency, API = Application Programming Interface, DNAm = DNA methylation, miRNA = microRNA, mRNA = messengerRNA, GWAS = Genome-wide association study, LD = Linkage disequilibrium, MAF = Minor allele frequency, SNP = Single nucleotide polymorphism, STR = Short tandem repeat, WGBS = Whole genome bisulfite sequencing, WGS = Whole genome sequencing

| **Reference** | **Attack type** | **Dataset type under attack** | **Information gained** | **Requirements for success** | **Public resources used** |
| --- | --- | --- | --- | --- | --- |
| Almadhoun, N., Ayday, E., & Ulusoy, Ö. (2020). Inference attacks against differentially private query results from genomic datasets including dependent tuples. Bioinformatics, 36(Supplement_1), i136-i145. | Inference and membership (Beacon attack) | Summary statistics (differentially-private). 1) for inference attack: AFs OR chi-square value of 100 SNPs on the same chromosome (chr22) 2) for membership attack: SNP AFs of 250 SNPs from a dataset of n=80 participants (including the target). | 1) SNP data of target (up to 100 SNPs), 2) membership of target in a dataset | For inference: Adversary has unlimited queries to beacon and knows that the target and family members of the target are contained in the beacon dataset. Adversary knows demographic information of target and family members (age, location of residence etc.,) and can use this information to query the beacon specifically for data that includes the target's relatives. For membership: Adversary knows target's genetic data (250 SNPs). | none |
| Ayoz, K., Ayday, E., & Cicek, A. E. (2021). Genome reconstruction attacks against genomic data-sharing beacons. In Proceedings on Privacy Enhancing Technologies. Privacy Enhancing Technologies Symposium (Vol. 2021, No. 3, p. 28). NIH Public Access. | Inference and membership (Beacon attack) | Summary statistics: Genomic beacon (GA4GH) (size n=50, 2-4 million SNPs), queried at different timepoints (prior and after inclusion of target) | 1) SNP data of target 2) membership of target in a dataset | Adversary knows that target is in a beacon, knows that target has been added to the beacon recently and has a snapshot of the beacon (responses to all queries) before the target was added. Adversary has information about target's visible characteristics (eye color, hair color etc.,). Adversary can pose unlimited queries to the beacon. | dbSNP (for population MAFs), OpenSNP (for SNP-SNP correlations and SNP-phenotype associations, e.g. eye color, hair color etc) |
| Backes, M., Berrang, P., Bieg, M., Eils, R., Herrmann, C., Humbert, M., & Lehmann, I. (2017, May). Identifying personal DNA methylation profiles by genotype inference. In 2017 IEEE Symposium on Security and Privacy (SP) (pp. 957-976). IEEE. | Inference (Linking DNA and DNAm) | Paired DNAm and SNPs. 13 meQTL pairs (highly informative SNPs plus associated methylated regions) were sufficient. Dataset size n=75 | Linkage of target's DNAm data to SNP data | SNP and DNAm dataset, both containing highly informative meQTL loci and target's data. Attacker knows either DNAm or SNPs of target individual (13 meQTL loci can be sufficient) and dataset is small (n=75) | dbSNP, paired DNAm and SNP data for identifying meQTLs or other public knowledge of meQTL loci |
| Backes, M., Berrang, P., Humbert, M., & Manoharan, P. (2016, October). Membership privacy in MicroRNA-based studies. In Proceedings of the 2016 ACM SIGSAC Conference on Computer and Communications Security (pp. 319-330). | Membership (Summary statistics) | Summary statistics: Mean miRNA expression values (848 miRNAs) | Membership of target in a dataset |  | Gene Expression Omnibus or ArrayExpress for miRNA reference population |
| Berrang, P., Humbert, M., Zhang, Y., Lehmann, I., Eils, R., & Backes, M. (2018, April). Dissecting privacy risks in biomedical data. In 2018 IEEE European Symposium on Security and Privacy (EuroS&P) (pp. 62-76). IEEE. | Inference (Linking DNA and DNAm) | Paired SNP and DNAm data (WGBS). Different subsets but maximally 31,586 pairs of SNPs and correlated methylated regions were used | SNPs from DNAm, DNAm from SNPs, as well as SNPs and DNAm of a child given SNPs and DNAm of the mother (and other way around), and DNAm at timepoint X to predict DNAm at timepoint Y. | Adversary knows DNAm or genetic data (SNP) from a relative of the target or from the target itself at a different point in time (e.g. DNAm data from another study) (for inference across kin or time) | dbSNP (for population MAFs), training set of DNAm and SNP pairs with ~30,000 eQTLs to train model, Kaviar database (for SNV population AFs) |
| Braun, R., Rowe, W., Schaefer, C., Zhang, J., & Buetow, K. (2009). Needles in the haystack: identifying individuals present in pooled genomic data. PLoS genetics, 5(10), e1000668. | Membership (Summary statistics) | Summary statistics: SNP AFs (up to 550,000 SNPs, multiple scenarios were tested) | Membership of target in a dataset | Adversary knows target's genetic data (50,000-550,000 SNPs) and has access to AFs of a suitable reference cohort (i.e. from the same population as the target) | none |
| Bu, D., Wang, X., & Tang, H. (2021). Haplotype-based membership inference from summary genomic data. Bioinformatics, 37(Supplement_1), i161-i168. | Membership (Summary statistics) | Summary statistics: SNP AFs OR genomic beacon (GA4GH) (SNP data from 1000 Genomes Project, restricted to chromosome 10) | Membership of target in a dataset | Adversary knows target's genetic data (all SNPs on one chromosome) | 1000 Genomes project (for haploblock structure in human genome) |
| Cai, R., Hao, Z., Winslett, M., Xiao, X., Yang, Y., Zhang, Z., & Zhou, S. (2015). Deterministic identification of specific individuals from GWAS results. Bioinformatics, 31(11), 1701-1707. | Membership (Summary statistics) | Summary statistics: SNP AFs, SNP-phenotype correlation and SNP-SNP correlation from GWAS. Data from 25 loci was sufficient. | Membership of target in a dataset and whether target was in case or control group | Adversary knows target's genetic data (25 SNPs) and has access to AFs of a suitable reference cohort (i.e. from the same population as the target) | none |
| Deznabi, I., Mobayen, M., Jafari, N., Tastan, O., & Ayday, E. (2017). An inference attack on genomic data using kinship, complex correlations, and phenotype information. IEEE/ACM transactions on computational biology and bioinformatics, 15(4), 1333-1343. | Inference (SNP imputation) | SNPs: 100 SNPs (all on one chromosome, from .vcf file) from multiple individuals that are related to a target individual | SNP data of target (50 SNPs) |  | 1000 Genomes Project and HapMap (to infer SNP higher order correlations and AFs) |
| Dyke, S. O., Cheung, W. A., Joly, Y., Ammerpohl, O., Lutsik, P., Rothstein, M. A., ... & Pastinen, T. (2015). Epigenome data release: a participant-centered approach to privacy protection. Genome biology, 16(1), 1-12. | Inference (Linking DNA and DNAm) | DNAm data: genome-wide (from WGBS and Illumina450k array) | SNP data of target | Adversary has access to target's DNAm data (any of the 50,000 CpGs that directly overlap a SNP) | NIH RoadMap Epigenomics WGBS samples for identification of SNP-resolving CpG sites |
| Edge, M. D., Algee-Hewitt, B. F., Pemberton, T. J., Li, J. Z., & Rosenberg, N. A. (2017). Linkage disequilibrium matches forensic genetic records to disjoint genomic marker sets. Proceedings of the National Academy of Sciences, 114(22), 5671-5676. | Inference (Linking STR and SNP) | Paired SNP (642,563 SNPs) and STR data (13 STRs). SNPs nearby STRs were used for the attack. | Linkage of target's SNP to STR record and vice versa | Adversary has access to target's genetic data (either SNP or CODIS STRs) and has access to a small to medium sized database (N<1000 in this study) containing the respective other genetic data (SNP or CODIS STRs) of the target | Beagle tool for genotype imputation, Human Genome Diversity Panel (as training data for learning SNP-STR associations) |
| Edge MD, Coop G. Attacks on genetic privacy via uploads to genealogical databases. Elife 2020; 9:1–24. | Inference (Geneaology attack) | GEDmatch database (>500,000 genome-wide SNPs), queried for geneaological matches | SNP data of public geneaology website users (GEDmatch) | Unlimited uploads to SNP based genetic geneaology database, which provides customers putative geneaological relatives as well as information about shared genomic regions with each putative relative (Only GEDmatch currently fulfills these requirements). | GEDmatch, Human Genome Diversity Project and 1000 Genomes project (to create artificial genomes for upload) |
| Erlich Y, Shor T, Pe'er I, et al (2018) Identity inference of genomic data using long-range familial searches. Science 362: 690–694 | Identity tracing (Geneaology attack) | SNPs: 700.000 (from raw genotype file) | Sensitive information of target (surname) | Adversary knows target's genetic data (700,000 SNPs), relatives of target have uploaded their genetic data on publicly accessible genetic geneaology databases, along with dentifying information (place of residence, age, surname) which allows triangulation of identity of the target. | GEDmatch |
| Fredrikson, M., Lantz, E., Jha, S., Lin, S., Page, D., & Ristenpart, T. (2014). Privacy in Pharmacogenetics: An {End-to-End} Case Study of Personalized Warfarin Dosing. In 23rd USENIX Security Symposium (USENIX Security 14) (pp. 17-32). | Inference (Model inversion) | Summary statistics: Linear model (differentially private) trained on a dataset (regression coefficients). The model predicts medication dose requirements for patients based on their age, height, weight, race, other medication and <10 SNPs. | SNP data of target | Adversary has access to the model, its performance, and its output for the target. In addition, adversary has access to either a) additional information about target that is used in the model (age, race, height, weight in this case) or b) distribution of these variables in the cohort | none |
| Gitschier, J. (2009). Inferential genotyping of Y chromosomes in Latter-Day Saints founders and comparison to Utah samples in the HapMap project. The American Journal of Human Genetics, 84(2), 251-258. | Identity tracing (Geneaology attack) | STRs: 17 Y-STRs | Sensitive information of target (surname) | Target is male, relatives of target have uploaded their genetic information on publicly accessible genetic geneaology databases, along with dentifying information (place of residence, age, surname) which allows triangulation of identity of the target. | SMGF, FamilySearch (genealogical registry) |
| Gymrek M, McGuire AL, Golan D, et al (2013) Identifying personal genomes by surname inference. Science 339: 321–324 | Identity tracing (Linking STR and SNP, genealogy attack) | STRs: 34 Y-STRs | Sensitive information of target (surname) | Target is male, relatives of target have uploaded their genetic information on publicly accessible genetic geneaology databases (>500.000 SNPs), along with dentifying information (place of residence, age, surname) which allows triangulation of identity of the target. | Ysearch, SMGF |
| Gürsoy, G., Lu, N., Wagner, S., & Gerstein, M. (2021). Recovering genotypes and phenotypes using allele-specific genes. Genome biology, 22(1), 1-9. | Inference (Linking DNA and RNA) | Summary statistics: List of allele-specific genes of individuals (can also be extracted from RNA expression data) | SNP data and sensitive information of target (disease phenotype) | None | GTEx (for SNP-gene expression associations), PsychENCODE (for genotype-phenotype associations) |
| Hagestedt, I., Zhang, Y., Humbert, M., Berrang, P., Haixu, T., XiaoFeng, W., & Backes, M. (2019). MBeacon: Privacy-preserving beacons for DNA methylation data. | Membership (Beacon attack) | Summary statistics: DNAm beacons | Membership of target in a dataset | Adversary knows target's DNAm data | DNAm levels (mean and standard deviation) of reference cohort |
| Harmanci, A., & Gerstein, M. (2016). Quantification of private information leakage from phenotype-genotype data: linking attacks. Nature methods, 13(3), 251-256. | Inference (Linking DNA and RNA) | Paired RNA data (gene expression values from RNAseq) and SNPs: >500,000 SNPs, dataset size n=421 | Linkage of target's mRNA record with corresponding SNP record (or other way around) | Adversary has access to a mRNA dataset (gene expression levels) and a genetic dataset (SNPs), both containing the target's data | GEUVADIS, GTEx (for genotype-expression correlations, i.e. eQTLs) |
| Harmanci, A., & Gerstein, M. (2018). Analysis of sensitive information leakage in functional genomics signal profiles through genomic deletions. Nature communications, 9(1), 1-10. | Inference (Linking DNA and RNA) | Paired structural variation data (deletions) and RNA data: Read depth signal profiles (RNAseq, ChIP-Seq) (Linkage successful with <100 variants) | SNP data and structural variation data of target (deletions) | Adversary has access to RNA raw data (signal profiles) and a genetic dataset (structural variants), both containing the target's data | none |
| He, Z., Yu, J., Li, J., Han, Q., Luo, G., & Li, Y. (2018). Inference attacks and controls on genotypes and phenotypes for individual genomic data. IEEE/ACM transactions on computational biology and bioinformatics, 17(3), 930-937. | Inference (Phenotype-genotype matching or prediction) | Paired SNPs: any number of known SNPs, and traits from an individual (for example 5 SNPs and 3 traits) | SNP data of target and sensitive information (phenotype, traits or predispositions) | Adversary knows target's genome and their relatives' genomes, as well as traits of target and relatives if available | GWAS catalog (for SNP-trait associations, statistical relations between SNPs and SNP frequencies) |
| Homer N, Szelinger S, Redman M, et al (2008) Resolving individuals contributing trace amounts of DNA to highly complex mixtures using high-density SNP genotyping microarrays. PLoS Genet 4: e1000167 | Membership (Summary statistics) | Summary statistics: SNP AFs | Membership of target in a dataset | Adversary knows target's genetic data (SNPs) and has access to AFs of a suitable reference cohort (i.e. from the same population as the target) | none |
| Humbert, M., Ayday, E., Hubaux, J. P., & Telenti, A. (2017). Quantifying interdependent risks in genomic privacy. ACM Transactions on Privacy and Security (TOPS), 20(1), 1-31. | Inference (Phenotype-genotype matching or prediction) | SNPs: 81,899 SNPs of relatives of a target individual | SNP data of target | Adversary knows genetic data of one or more family members of target and knows familial relationship | Population MAFs and SNP LD values |
| Humbert, M., Ayday, E., Hubaux, J. P., & Telenti, A. (2013, November). Addressing the concerns of the Lacks family: quantification of kin genomic privacy. In Proceedings of the 2013 ACM SIGSAC conference on Computer & communications security (pp. 1141-1152). | Inference  (SNP imputation) | SNPs: examples of 50, 100 and 80,000 randomly selected SNPs, all from chromosome 1, from relatives of the target | SNP data of target | Adversary knows genetic data of relatives and knows relation to target | Population MAFs and LDs, social networks or geneaology websites (for familial relationships) |
| Humbert, M., Huguenin, K., Hugonot, J., Ayday, E., & Hubaux, J. P. (2015). De-anonymizing genomic databases using phenotypic traits. Proceedings on Privacy Enhancing Technologies, 2015(2). | Inference (Phenotype-genotype matching or prediction) | SNPs: 20 for unsupervised, 34 for supervised | With successful linkage of phenotypic traits and individual's genome, it allows inference from genome | Adversary knows target's genetic data and phenotypic traits, adversary would also need to know that individual is part of the dataset | SNPedia |
| Hae KI, Gamazon ER, Nicolae DL, et al (2012) On sharing quantitative trait GWAS results in an era of multiple-omics data and the limits of genomic privacy. Am J Hum Genet 90: 591–598 | Membership (Summary statistics) | Summary statistics: SNP regression coefficients from GWAS | Membership of target in a dataset | Adversary knows target individual's genome (SNPs) |  |
| Kim, J., Edge, M. D., Algee-Hewitt, B. F., Li, J. Z., & Rosenberg, N. A. (2018). Statistical detection of relatives typed with disjoint forensic and biomedical loci. Cell, 175(3), 848-858. | Inference (Linking STR and SNP) | Paired SNPs: 642,563 SNPs, and STRs: 13 CODIS STRs | Membership of relatives of a target in a dataset that contains another type of genetic markers (STRs vs. SNPs) | Adversary knows target's genetic data (CODIS STRs or SNPs) | Human Genome Diversity Panel (for learning STR-SNP associations) |
| Lippert C, Sabatini R, Maher MC, et al (2017) Identification of individuals by trait prediction using whole-genome sequencing data. Proc Natl Acad Sci USA 114: 10166–10171 | Inference (Phenotype-genotype matching or prediction) | SNPs: > 6 million SNPs (from WGS) | Sensitive information of target (phenotypic traits such as sex, age, ethnicity, height) | Adversary knows target's genome and to auxillary identified data that can be used for matching | Genotype-phenotype associations |
| Malin, B., & Sweeney, L. (2004). How (not) to protect genomic data privacy in a distributed network: using trail re-identification to evaluate and design anonymity protection systems. Journal of biomedical informatics, 37(3), 179-192. | Identity tracing | Hospital discharge data and genetic data (monogenetic disease causing genetic alterations) of the target from multiple hospitals (data trails) | Linkage of genetic data to sensitive information of target (name and demographics from hospital data) | Target has a genetic disease which allows linkage of genetic data with health records (e.g. cystic fibrosis, Huntington's) | none |
| Ney, P., Ceze, L., & Kohno, T. (2020). Genotype Extraction and False Relative Attacks: Security Risks to Third-Party Genetic Genealogy Services Beyond Identity Inference. In NDSS. | Inference (Geneaology attack) | Public genealogy database (GEDmatch) | Infer genetic information of individuals in the database; infer false identity relationships | Adversary knows target is in database, knows which genotyping kit the target has used, has unlimited queries to the database and receives genetic information about matches | Population MAFs |
| Nyholt, D. R., Yu, C. E., & Visscher, P. M. (2009). On Jim Watson's APOE status: genetic information is hard to hide. European Journal of Human Genetics, 17(2), 147-149. | Inference (Phenotype-genotype matching or prediction) | SNPs: 144 SNPs (from WGS) | SNP data of target and sensitive information of target (disease risk: late-onset Alzheimer's disease) | SNPs are directly or indirectly (through linkage disequilibrium) associated with sensitive phenotype | HapMap (CEU)-phased haplotype data and previous publications on genetics of Alzheimer's disease |
| Philibert, R. A., Terry, N., Erwin, C., Philibert, W. J., Beach, S. R., & Brody, G. H. (2014). Methylation array data can simultaneously identify individuals and convey protected health information: an unrecognized ethical concern. Clinical epigenetics, 6(1), 1-6. | Inference  (SNP imputation) | DNAm data: low-resolution genome-wide DNAm data (microarray data with 485,577 beta values binned in 3 bins (low, medium, high methylation)) | SNP data (1,069 SNPs) of target and sensitive information of target (tobacco consumption) |  |  |
| Raisaro, J. L., Tramer, F., Ji, Z., Bu, D., Zhao, Y., Carey, K., ... & Hubaux, J. P. (2017). Addressing Beacon re-identification attacks: quantification and mitigation of privacy risks. Journal of the American Medical Informatics Association, 24(4), 799-805. | Membership (Beacon attack) | Summary statistics: Genomic beacons (GA4GH) (beacon size n=206), restricted to AFs of SNPs on chromosome 10. 3-36 queries. | Membership of target in a dataset | Adversary knows target's genome (SNPs) and has access to AFs of a suitable reference cohort (i.e. from the same population as the target) | MAFs of reference cohort |
| Sankararaman, S., Obozinski, G., Jordan, M. I., & Halperin, E. (2009). Genomic privacy and limits of individual detection in a pool. Nature genetics, 41(9), 965-967. | Membership (Summary statistics) | Summary statistics: MAFs of various numbers of SNPs (1,000; 10,000; 33,138 SNPs, selected from ~500,000 SNPs (microarray data), dataset size n=1,000 | Membership of target in a dataset | Adversary has access to target's genome (SNPs) and has access to AFs of a suitable reference cohort (i.e. from the same population as the target). | MAFs of reference cohort |
| Schadt EE, Woo S, Hao K (2012) Bayesian method to predict individual SNP genotypes from gene expression data. Nat Genet 44: 603–608 | Inference (Linking DNA and RNA) | Paired RNA data: 40,000 transcripts (from microarray) and SNPs: 574,000 SNPs | SNP data of target | For linkage: adversary has access to RNA dataset (gene expression levels) and genetic dataset (SNPs), both containing the target's data | gene expression datasets that are accessible in public databases, such as those provided by the International HapMap Project and the GEO database |
| Sero, D., Zaidi, A., Li, J., White, J. D., Zarzar, T. B. G., Marazita, M. L., ... & Claes, P. (2019). Facial recognition from DNA using face-to-DNA classifiers. Nature communications, 10(1), 2557. | Inference (Phenotype-genotype matching or prediction) | SNPs: > 500,000 SNPs (Whole genome sequencing, HapMap) | Sensitive information of target (phenotypic traits: prediction of a 3D image of a person's face) and linkage of target's genetic data and 3D image of face |  | none, paired genotype and 3D facial images for training were taken from a study which is not publicly available |
| Shringarpure, S. S., & Bustamante, C. D. (2015). Privacy risks from genomic data-sharing beacons. The American Journal of Human Genetics, 97(5), 631-646. | Inference and membership (Beacon attack) | Summary statistics: Genomic beacons (GA4GH): 1000 queries | Membership of target in a dataset | Adversary has access to target's genome or genome of their relatives (in the scenario, the attacker has access to VCF file listing all the SNP positions at which the query individual has an alternate allele and the genotype calls at the corresponding positions) | None |
| Venkatesaramani, R., Malin, B. A., & Vorobeychik, Y. (2021). Re-identification of individuals in genomic datasets using public face images. Science advances, 7(47), eabg3296. | Inference (Phenotype-genotype matching or prediction) | SNPs: > 500,000 SNPs (23andMe-sequenced genetic data on OpenSNP) | Linkage between target's genetic data and face image | Adversary has access to a high quality image of the target's face and knows that the target is part of the genetic dataset. | Genome-photo pairs from OpenSNP for training |
| Nora von Thenen, Erman Ayday, A Ercument Cicek, Re-identification of individuals in genomic data-sharing beacons via allele inference, Bioinformatics, Volume 35, Issue 3, 01 February 2019, Pages 365–371, https://doi.org/10.1093/bioinformatics/bty643 | Membership and inference (Beacon attack) | Summary statistics: Genomic beacons (GA4GH), dataset size n=65. 5-60,000 queries | Membership of target in a dataset and SNP data of target | Adversary knows target's genome (SNPs) and has access to AFs and LDs of a suitable reference cohort (i.e. from the same population as the target). | Hapmap, 1000 Genomes project (for SNP-SNP associations), MAFs and LDs of reference cohort |
| Wang, Y., Wen, J., Wu, X., & Shi, X. (2016). Infringement of individual privacy via mining differentially private GWAS statistics. In Big Data Computing and Communications: Second International Conference, BigCom 2016, Shenyang, China, July 29-31, 2016. Proceedings 2 (pp. 355-366). Springer International Publishing. | Inference (Summary statistics) | Summary statistics: trait-associated SNPs, odds ratio, p-value from GWAS, dataset size n=85 (1000 Genomes data) | Sensitive information of target (phenotypic traits and disease risk) | Adversary knows target's genome (< 1000 SNPs) | GWAS catalog (for SNP-trait associations) |
| Wang, Y., Wu, X., & Shi, X. (2013, December). Using aggregate human genome data for individual identification. In 2013 IEEE International Conference on Bioinformatics and Biomedicine (pp. 410-415). IEEE. | Inference (Phenotype-genotype matching or prediction) | SNPs: > 500,000 SNPs (1000 Genomes data), 9 SNPs used for trait inference | Sensitive information of target (six phenotypic traits such as height, eye color and presence of diseases) and linkage of target to a genetic record based on known phenotypic traits |  | GWAS catalog (for SNP-trait associations) |
| Wang, R., Li, Y. F., Wang, X., Tang, H., & Zhou, X. (2009, November). Learning your identity and disease from research papers: information leaks in genome wide association study. In Proceedings of the 16th ACM conference on Computer and communications security (pp. 534-544). | Inference and membership (Summary statistics) | Summary statistics: Correlations (r-values) between SNPs and associated p-values from GWAS | Membership of target in a dataset | Adversary knows target individual's genome (SNPs) | none |
